# The LIFR-targeting small molecules EC330/EC359 are potent ferroptosis inducers

**DOI:** 10.1016/j.gendis.2022.10.016

**Published:** 2022-11-14

**Authors:** Chang-Zhou Feng, Ning-Zhe Li, Xi-Bo Hu, Yin-Yin Xie, Qiu-Hua Huang, Jianming Zhang, Zhu Chen, Sai-Juan Chen, Fudi Wang, Xiao-Jian Sun

**Affiliations:** a Shanghai Institute of Hematology, State Key Laboratory of Medical Genomics, National Research Center for Translational Medicine at Shanghai, Ruijin Hospital Affiliated to Shanghai Jiao Tong University School of Medicine, Shanghai 200025, China; b School of Life Sciences & Biotechnology, Shanghai Jiao Tong University, Shanghai 200240, China; c The First Affiliated Hospital, School of Public Health, Institute of Translational Medicine, Zhejiang University School of Medicine, Hangzhou, Zhejiang 310058, China; d The First Affiliated Hospital, Hengyang Medical School, University of South China, Hengyang, Hunan 421001, China

Ferroptosis is a form of regulated cell death characterized by iron-dependent overaccumulation of lipid peroxides, which causes membrane damage and cell lysis. Ferroptosis can be prevented by cellular antioxidant mechanisms such as glutathione peroxidase 4 (GPX4)-mediated elimination of the lipid peroxides at the cost of glutathione (GSH). As the rate-limiting step of GSH synthesis, the availability of intracellular cystine is controlled by the cell membrane-located cystine/glutamate antiporter system x_c_^−^. Indeed, the initially identified small molecule inducers of ferroptosis, RSL3 and erastin, turned out to be inhibitors of GPX4 and system x_c_^−^, respectively.^1^^,^^2^

Since originally being discovered as a property of *RAS*-mutant cancer cells, ferroptosis has been shown to be regulated by several oncogenes and tumor suppressors. LIFR, the transmembrane receptor of the pleiotropic cytokine leukemia inhibitory factor (LIF), has recently been shown to be required for ferroptosis, as loss of *Lifr* gene in mouse liver tumor confers resistance to ferroptosis.^3^ Interestingly, in the present study, we found that the LIFR-targeting small molecules, EC330 and EC359, are potent ferroptosis inducers. EC330/EC359 are steroidal molecules designed to bind to LIFR at one (the “site 3”) of two interacting interfaces within the ligand-receptor complex (Fig. 1A).^4^ EC330/EC359 can suppress LIF-induced activation of STAT3 and AKT pathways and cause cell death correlated with the expression levels of LIF and LIFR in the cells.^4^ However, it is unclear whether EC330/EC359 could inhibit the whole biological functions of LIFR, and the EC330/EC359-induced cell death has not been fully characterized. In this regard, given the recently reported positive role of LIFR in ferroptosis,^3^ our identification of EC330/EC359 as ferroptosis inducers (instead of inhibitors) suggests a possibility that EC330/EC359 induce ferroptosis through a mechanism(s) beyond simply acting as inhibitors of LIFR.

**Figure 1:**
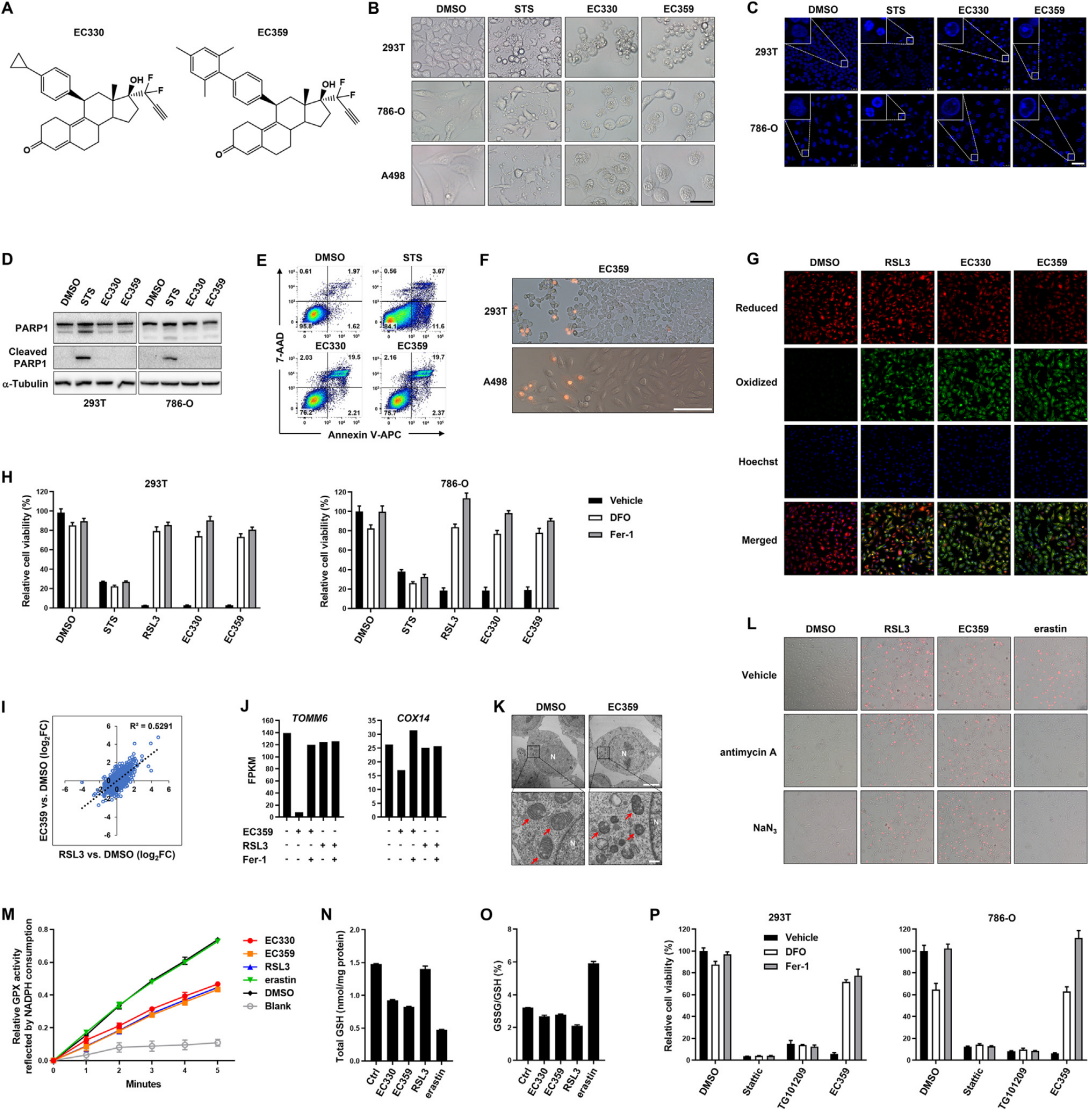
Characterization of EC330/EC359-induced ferroptosis. **(A)** Chemical structures of EC330 and EC359. **(B)** Morphological changes of the embryonic kidney 293T cells and renal carcinoma 786-O and A498 cells upon treatment with STS, EC330, and EC359 under a light microscope. Solvent DMSO was added to the control wells. Scale bar = 50 μM. **(C)** Hoechst staining of the nuclei of the treated cells. Magnified areas are shown at the upper-left corners. Scale bar = 50 μM. **(D)** Immunoblot of PARP1 and its cleaved form in the treated cells. α-Tubulin was used as a loading control. **(E)** Flow cytometry analysis of cell death with Annexin V and 7-AAD. **(F)** PI staining the cells in culture, showing representative snapshots of wave-like propagation of cell death. Scale bar = 500 μM. **(G)** Confocal microscopy images showing the production of lipid peroxidation as sensed by C11-BODIPY. Red and green fluorescent colors reflect reduced and oxidized lipids, respectively, and blue indicates Hoechst staining of the nuclei. **(H)** Effects of DFO and Fer-1 on the cell death induced by indicated drugs. **(I)** RNA-seq analysis of the EC359- and RSL3-treated 293T cells, compared with the control cells (DMSO added). **(J)***TOMM6* and *COX14*, two genes relevant to mitochondrial functions, are presented as examples of a small group of genes that could be regulated by EC359 but not RSL3. **(K)** Representative TEM images of the 293T cells treated with EC359, compared with control cells. Red arrows denote mitochondria. N, nucleus. Scale bars in the upper and lower panels indicate 5 μM and 500 nM, respectively. **(L)** Effects of the ETC inhibitors antimycin A and NaN_3_ on the ferroptosis induced by RSL3, EC359, and erastin. **(M)** Cellular GPX activity assay by measuring NADPH consumption, showing decreased GPX activities in the EC330/EC359-treated cells. **(N)** Measurement of total GSH levels in the cells. **(O)** Calculation of GSSG-to-GSH ratio in the cells. **(P)** Cell death induced by the JAK/STAT3 inhibitors Stattic and TG101209 could not be rescued by DFO or Fer-1. Rescue of EC359-induced ferroptosis was performed as a positive control. In panels (H, M−P), data are presented as means ± SD of triplicate experiments.

We found that EC330/EC359-treatment of several types of human cells caused rapid nonapoptotic cell death with characteristics of ferroptosis, in contrast with apoptosis of the cells induced by staurosporine (STS). A clear morphological change was that the cytoplasm of the EC330/EC359-treated cells swelled dramatically and the nuclei remained intact, whereas the STS treatment caused cell shrinkage, membrane blebbing, and generation of apoptotic bodies (Fig. 1B). Lactate dehydrogenase (LDH) release analysis showed that the cell death induced by EC330/EC359 started as early as 4 h. Consistent with previous observations,^4^ these cells showed different sensitivities correlated with their LIF/LIFR expression levels (Fig. S1). Staining the nuclei with Hoechst showed that chromatin condensation, a hallmark of apoptosis, occurred in the STS- but not EC330/EC359-treated cells (Fig. 1C). Immunoblot analysis showed that only STS induced cleavage of PARP1 by apoptotic caspases (Fig. 1D). Moreover, flow cytometry analysis of the cells with Annexin V and 7-aminoactinomycin D (7-AAD) showed that unlike STS causing Annexin V^+^/7-AAD^−^ early apoptotic cells in a short treatment, the EC330/EC359-treated cells directly became Annexin V^+^/7-AAD^+^, suggesting an early rupture of the cell membrane that allowed 7-AAD permeating into the cells (Fig. 1E). Related to this notion, staining the EC330/EC359-treated cells in culture with propidium iodide (PI) revealed an intercellular propagation of cell death between neighboring cells (Fig. 1F), representing a feature of ferroptosis that involves cell swelling and solute exchange through the ruptured cell membrane.

We then analyzed the cells with C11-BODIPY, a dye that can sense lipid peroxidation. The results showed that the EC330/EC359 treatment caused a significant lipid peroxidation comparable with the effect of RSL3 (Fig. 1G; Fig. S2). Accordingly, co-treatment with either the iron chelator deferoxamine (DFO) or the lipid peroxide scavenger ferrostatin-1 (Fer-1) significantly suppressed EC330/EC359-induced cell death (Fig. 1H). In contrast, this cell death could not be inhibited by the pan-caspase inhibitor z-VAD-fmk or the necroptosis inhibitor necrostatin-1 (Nec-1) (Fig. S3).

To understand the molecular mechanism, we performed RNA-seq analysis of the EC359- and RSL3-treated cells, as well as those co-treated with Fer-1. The results showed that the EC359 treatment leads to a gene expression profile highly resembling that of RSL3 (Fig. 1I), thus validating the molecular essence of ferroptosis. Meanwhile, EC359 specifically altered a small group of genes that were not affected by RSL3. Several of these genes (e.g., *TOMM6* and *COX14*) encode mitochondrial-membrane-located proteins that are important for mitochondrial protein synthesis, translocation, and function (Fig. 1J). This finding suggests possible defects of the EC359-treated cells in their mitochondrial membrane integrity and function. Indeed, transmission electron microscopy (TEM) imaging of the EC359-treated cells demonstrated shrunken mitochondria and loss of cristae (Fig. 1K).

Notably, previous studies have shown that inhibition of the electron transport chain (ETC) in mitochondria can suppress ferroptosis, but a prerequisite for this effect is high cellular GPX4 activity to counteract lipid peroxidation.^5^ To determine whether the EC359-induced ferroptosis could be suppressed by ETC inhibition, we applied two ETC inhibitors, antimycin A and NaN_3_, in the EC359-, RSL3-, and erastin-treating cells. The results showed that these ETC inhibitors could only suppress ferroptosis induced by erastin, but not EC359 or RSL3 (Fig. 1L). Therefore, we speculated that the EC359-treated cells may have low GPX4 activity.

We next examined the intracellular GPX activity using a reaction system in which the GPX-catalyzed GSH peroxidation can be reflected by the consumption of NADPH. The results showed that EC330/EC359 dramatically decreased the intracellular GPX activity to a comparable level as the RSL3-treated cells (Fig. 1M). Intracellular GSH analysis showed that EC330/EC359 also decreased GSH level, which somewhat resembled the effect of erastin but not RSL3 (Fig. 1N); nevertheless, as GPX4 was inactive in these cells, the EC330/EC359-induced GSH depletion was not correlated with an extraordinarily high ratio of oxidized-to-reduced GSH (i.e., GSSG/GSH) as achieved by erastin (Fig. 1O). Therefore, the EC330/EC359 treatment results in both GPX4 inactivation and GSH depletion, and these two effects may jointly disable the cells to eliminate the toxic lipid peroxides, and eventually lead to ferroptosis.

Lastly, we investigated whether the STAT3 and AKT signaling pathways downstream of LIFR could contribute to the EC330/EC359-induced ferroptosis. We found that, although these pathways could indeed be inhibited by EC330/EC359, inhibition of these pathways personally induced non-ferroptotic cell death, as the cells treated with the JAK/STAT3 inhibitors Stattic or TG101209 could not be rescued by ferroptosis inhibitors DFO or Fer-1 (Fig. 1P). We also tested the human breast cancer cell line BT-549, which was previously used to study the signaling pathways inhibited by EC359 and the subsequent cell death.^4^ We found that the BT-549 cells similarly underwent ferroptosis rather than apoptosis upon EC330/EC359 treatment, because they were largely rescued by DFO and Fer-1 (Fig. S4). These results suggest that the EC330/EC359-induced ferroptosis is independent of these downstream pathways. Meanwhile, the LIFR-NFκB-LCN2 axis recently discovered in mouse liver tumor cells^3^ might not explain the EC330/EC359-induced ferroptosis either, because LCN2 is not detectable in the herein used cell lines (data not shown).

Taken together, these results reveal an unexpected role of the LIFR-targeting small molecules EC330/EC359 as potent ferroptosis inducers. In combination with previous studies, our results suggest that there could be an unrecognized mechanism of LIFR-associated ferroptosis or an unknown target for EC330/EC359 to induce ferroptosis. On one hand, although EC330/EC359 is designed to directly bind to LIFR, there is no experimental evidence to show that they can completely block the ligand-receptor interaction or inhibit all the functions of LIFR. There is a chance that EC330/EC359 may even enhance a function of LIFR or confer LIFR a new/alternative function. On the other hand, EC330/EC359 may have another target that plays an important role in ferroptosis. Given the high similarities between the EC330/EC359- and RSL3-treated cells in their morphology and molecular features, it merits further investigation to determine whether GPX4 could serve as a direct or indirect target of EC330/EC359.

## Conflict of interests

The authors declare no conflict of interests.

## Funding

This work was supported by the National Key R&D Plan of China (2018YFA0107802) (to X-JS), the 10.13039/501100001809National Natural Science Foundation of China (NSFC) General Program (No. 81670094 and 81470316) (to X-JS), the Shanghai Municipal Education Commission-Gaofeng Clinical Medicine (China) (No. 20152506) (to X-JS), Shanghai Collaborative Innovation Program on Regenerative Medicine and Stem Cell Research (China) (No. 2019CXJQ01) (to S-JC and X-JS), the 10.13039/100001384Samuel Waxman Cancer Research Foundation, and the Shanghai Guangci Translational Medical Research Development Foundation. X-JS was supported by the 1000 Talents Program for Young Scholars (China).

## Data availability statement

RNA-seq data were deposited in the GEO database with accession No. GSE201493.
