# Supplementary material for: The LIFR-targeting small molecules EC330/EC359 are potent ferroptosis inducers
Source: Genes Dis. 2022 Nov 14;10(3):735–8. doi: 10.1016/j.gendis.2022.10.016 (PMC10308161; doi:10.1016/j.gendis.2022.10.016)
Supplement: Multimedia component 1 [file mmc1.docx]

**Supplementary figures**





**Supplementary Figure S1.** Rapid cell death induced by EC359, and the different sensitivities of the cells that are correlated with the expression levels of LIF and LIFR. (**A, B**) Representative LDH release experiments using different concentrations of EC359 on 293T and 786-O cells. (**C**) RT-qPCR analysis of the expression levels of *LIF* and *LIFR* mRNAs in 293T and 786-O cells. gp130 (encoded by the *IL6ST* gene) is another subunit of the LIF receptor complex. Three other cytokines known to bind the LIFR/gp130 receptor complex, including cardiotrophin 1 (CTF1), ciliary neurotrophic factor (CNTF), and oncostatin M (OSM), were also analyzed, and they showed minimum expression in these cells. The expression levels were all normalized to *GAPDH*. In (A-C), data are presented as means ± SD of triplicate experiments


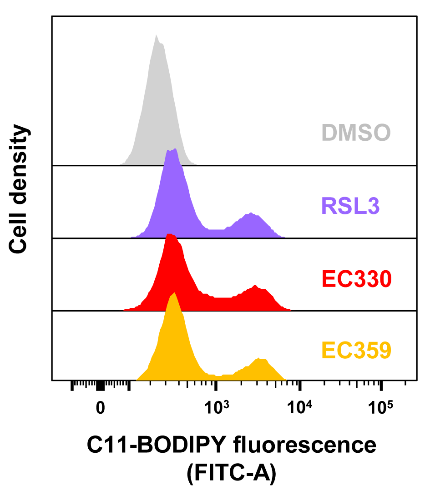


**Supplementary Figure S2.** Flow cytometry analysis of the EC330/EC359-treated cells that were stained with the lipid peroxidation sensor C11-BODIPY. The ferroptotic cell induced by RSL3 was used as a positive control. The fluorescein isothiocyanate (FITC)-A channel was used to detect the green fluorescence of C11-BODIPY.





**Supplementary Figure S3.** Lack of rescue effect of z-VAD-fmk and Nec-1 on the EC330/EC359-induced cell death. (**A**) Relative cell viabilities of 293T cells treated with indicated drugs and their combinations were determined by CCK-8 assay. Solvents of the drugs were added in the control wells. Data are presented as means ± SD of three separate cultures and reactions. (**B**) Similar results were observed in the experiments with 786-O cells.

**

**

**Supplementary Figure S4.** Effect of EC330/EC359 on BT-549 cells. Note that the BT-549 cells undergo ferroptosis rather than apoptosis upon EC330/EC359 treatment, because they can be largely rescued by DFO and Fer-1. In contrast, apoptosis of the BT-549 cells induced by staurosporine (STS) could not be affected by DFO or Fer-1. Data are presented as means ± SD of three separate cultures and reactions. Notably, the ferroptosis of BT-549 cells upon EC359 treatment was likely overlooked in the previous studies because there was not an apoptosis inducer being used as a control in those studies, and their Annexin V staining of the cells could not distinguish between apoptosis and ferroptosis.

**Supplementary materials and methods**

**Cell culture and chemical treatment**

Human embryonical kidney cell line 293T, and renal cell carcinoma cell lines (786-O and A498) and breast cancer cell line (BT-549) were cultured in DMEM (Gibco, C12430500CP) and RPMI 1640 medium (Gibco, C11875500CP), respectively, under standard condition (37°C and 5% CO2). Both media were supplemented with 10% FBS (Gibco, 16000-044) and penicillin/streptomycin (100 U/mL). The cells were seeded in 12-well plates, and treated with STS (Staurosporine; MCE, HY-15141), EC330 (Topscience, T7293) and EC359 (MCE, HY-120142) for 4 h when the cells were 70-90% confluent. DMSO was used as a negative control. Other chemical reagents used for this study were listed as follow: 1S,3R-RSL3 (Topscience, T3646), Stattic (Topscience, T6308), TG101209 (Topscience, T3065), Ferrostatin-1 (Topscience, T6500), Deferoxamine (Topscience, T1637), z-VAD-fmk (Topscience, T6013), Necrostatin-1 (Topscience, T1847), erastin (Selleck, S7242), antimycin A (Maokang, MS0070) and NaN3 (Sigma, S2002).

**Lactate dehydrogenase (LDH) release assay**

The LDH-Glo Cytotoxicity Assay Kit (Promega, J2380) was used for the LDH release assay according to the manufacturer's instructions. The cells were seeded in 12-well plates and cultured overnight prior to the drug treatment. Cell-free cultures were set as negative control. After treatment, the samples and LDH detection reagents were added at a 1:1 ratio into a 384-well plate and incubated for 40 min, then the luminescence signal was recorded using Varioskan Flash microplate reader (Thermo Scientific).

**RT-qPCR**

Total RNAs were extracted from harvested cells using TRIzol reagent (Invitrogen, 15596018) according to the manufacturer’s instructions. 1 µg RNA was reverse transcribed with PrimeScript RT Reagent Kit with gDNA Eraser (Takara, RR047B). RT-qPCR analysis was performed on the ViiA 7 Real-Time PCR System using the SYBR Premix Ex Taq Kit (TAKARA, RR820A). The relative mRNA expressions were calculated by the ΔΔCt method and normalized to the internal control gene *GAPD*H. Sequences of primers used for RT-qPCR were listed in the table below.

| **Primer** | **Sequence (5’ to 3’)** |
| --- | --- |
| *LIF*-qPCR-F | CCAACGTGACGGACTTCCC |
| *LIF*-qPCR-R | TACACGACTATGCGGTACAGC |
| *LIFR*-qPCR-F | TGGAACGACAGGGGTTCAGT |
| *LIFR*-qPCR-R | GAGTTGTGTTGTGGGTCACTAA |
| *gp130*-qPCR-F | CGGACAGCTTGAACAGAATGT |
| *gp130*-qPCR-R | ACCATCCCACTCACACCTCA |
| *CTF1*-qPCR-F | AGACCCCCAGACTGATTCCTC |
| *CTF1*-qPCR-R | AGCTGCACATATTCCTGGAGC |
| *CNTF*-qPCR-F | ACAGAGCATTCACCGCTGAC |
| *CNTF*-qPCR-R | TCAGGTCTGAACGAATCTTCCTT |
| *OSM*-qPCR-F | CACAGACTGGCCGACTTAGAG |
| *OSM*-qPCR-R | AGTCCTCGATGTTCAGCCCA |
| *GAPDH*-qPCR-F | GGAGCGAGATCCCTCCAAAAT |
| *GAPDH*-qPCR-R | GGCTGTTGTCATACTTCTCATGG |

**Hoechst staining**

Cells were washed once with culture medium and stained with Hoechst 33342 (Invitrogen, H3570) for 15 min. Then the stained cells were washed twice with culture medium and mounted for fluorescence imaging under a confocal microscopy (Leica SP8).

**Immunoblot**

Cells were collected with a cell scraper, washed with PBS, and lysed and boiled in 1x SDS loading buffer (Beyotime, P0015L) containing protease (Bimake, B14001) and phosphatase (Bimake; B15001) inhibitor cocktail. Antibodies for PARP1 (Cell Signaling technology, 9542), Cleaved PARP1 (Asp214) (D64E10) (Cell Signaling technology, 5625) and α-Tubulin (Proteintech, 11224-1-AP) were used for the immunoblot analysis. α-Tubulin was used as the loading control.

**Flow cytometry analysis with Annexin V and 7-amino-actinomycin D (7AAD)**

Cells were collected by trypsinization and washed twice with PBS, then stained with APC-conjugated Annexin V (Biosciences, 550474) and 7-amino-actinomycin D (7AAD) (Biolegend, 420404) for 20 min at 4 °C in darkroom. Then, the cells were resuspended with 200 μL binding buffer. Fluorescence intensity was measured by the BD LSRFortessa cell analyzer and the results was analyzed with the FlowJo software.

**Propidium iodide (PI) staining**

Cells were stained with PI (50 μg/mL) for 20 min and their fluorescent images were captured with a fluorescent microscopy (Olympus-CKX53).

**Lipid peroxidation detection**

BODIPY 581/591 C11 (Invitrogen, D3861) was used as a probe to detect the levels of lipid peroxidation. The cells in culture were incubated with BODIPY 581/591 C11 for 1 h and washed once with culture medium. For fluorescent imaging, the cells were further stained with Hoechst 33342 for 15 min, washed twice and subjected to fluorescent imaging with Leica SP8 confocal microscopy. For flow cytometry analysis, the cells were collected by trypsinization, washed twice with 1x PBS (containing 1% FBS), and then subjected to flow cytometry analyzer to measure the fluorescence intensity and analyzed with the FlowJo software.

**Cell viability assay**

Cell viability was determined using the Cell Counting kit-8 (CCK-8) (Bimake, B34302). Cells were seeded in 96-well culture plates at low density. After incubation for 12 h, 10 μL of culture media supplemented with indicated compounds were added to the cells and incubated for 48 h. Then, the absorbance at 450 nm was measured according to the manufacturer’s instructions using a microplate reader.

**RNA-Seq analysis**

Total RNA was extracted using TRIzol reagent according to the manufacturer’s instructions. Libraries were generated using the TruSeq RNA preparation kit (KAPA), CASAVA version 1.8 (Illumina), and sequenced with Illumina Navoseq 6000. Hisat2 was used to align the reads to GRCh38 and StringTie was used to quantify gene and isoform abundances. R statistical package software was utilized for differential expression analysis. RNA-seq data were deposited in the GEO database (accession no. GSE201493).

**Transmission electron microscopy (TEM)**

To visualize cellular ultrastructural changes, the cells were washed twice with serum-free medium and fixed with 2.5% glutaraldehyde in 0.1 M sodium phosphate buffer pH 7.4 for 2 h at 4°C. After dehydration, infiltration and embedding, samples were subjected to ultrathin sections using the Leica EM UC7. The ultrathin sections were stained with lead citrate and examined in Hitachi H-7650 electron microscopy.

**Glutathione peroxidase (GPX) activity assay**

The GPX activity was determined by measuring the nicotinamide adenine dinucleotide phosphate (NADPH) consumption using the Cellular Glutathione Peroxidase Assay Kit (Beyotime, S0056). The cells were collected and lysed according to the manufacturer’s recommendations and the OD value at 340 nm was measured using a microplate reader at 25°C.

**GSH and GSSG quantification**

The GSH and GSSG Assay Kit (Beyotime, S0053) was used to determine the levels of GSH and GSSG following the manufacturer’s protocol. Cells were collected and washed with 1× PBS at 4°C. After counting and weighing, the cell pellets were lysed with de-protein buffer, frozen and thawed in liquid nitrogen and 37°C water bath three times, and then centrifuged at 10000 xg for 10 min at 4°C. The supernatant was mixed with GSH assay reagents and incubated at 25°C for 5 min before addition of NADPH. The absorbance of each sample was measured at 412 nm by a microplate reader. Total GSH and GSSG were quantified by standard curve, and the GSSG-to-GSH ratio was calculated and used for analysis of redox status in the cells.

**Statistical analysis**

For the RT-qPCR, LDH release assay, cell viability, GSH and GSSG experiments, the data were presented as means ± SD of triplicate experiments. Student unpaired two-tailed t-test was used for most statistical analysis with the GraphPad Prism software.
